# Using PRRSV-Resilient Sows Improve Performance in Endemic Infected Farms with Recurrent Outbreaks

**DOI:** 10.3390/ani11030740

**Published:** 2021-03-08

**Authors:** Gloria Abella, Adela Pagès-Bernaus, Joan Estany, Ramona Natacha Pena, Lorenzo Fraile, Lluis Miquel Plà-Aragonés

**Affiliations:** 1 Department of Animal Science, University of Lleida, 25198 Lleida, Spain; gabellafalco@gmail.com (G.A.); jestany@ca.udl.cat (J.E.); romi.pena@udl.cat (R.N.P.); 2 Department of Mathematics, University of Lleida, 25001 Lleida, Spain; adela.pages@matematica.udl.cat (A.P.-B.); lmpla@matematica.udl.cat (L.M.P.-A.); 3 Department of Business Administration, University of Lleida, 25001 Lleida, Spain; 4 AGROTECNIO CERCA Center, 25198 Lleida, Spain

**Keywords:** PRRSV, resilient, susceptible, sow, production performance, economic performance

## Abstract

**Simple Summary:**

Porcine reproductive and respiratory syndrome (PRRS) is a viral disease responsible for huge economic losses to the pig industry. The selection of PRRSV resilient sows has been proposed as a strategy to control this disease. A simulation model was developed to test the differences in reproductive performance and economic outcome of resilient or susceptible sows under farm PRRSV endemic conditions with or without recurrent PRRSV outbreaks. The data from phenotyped sows came from a PRRSV-positive farm with 1500 sows that suffered a PRRSV outbreak that lasted 24 weeks within three years. The reproductive parameters were generally better for resilient than for susceptible sows in PRRSV-positive farms suffering recurrent PRRSV outbreaks. Consequently, the piglet production cost was lower for resilient than for susceptible sows in any condition but showed only significant differences in PRRSV endemic farms suffering recurrent outbreaks. Finally, the annual gross margin by sow is significantly better for resilient than for susceptible sows under endemic conditions with or without recurrent outbreaks. Thus, the selection of PRRSV resilient sows is always a profitable approach for producers supporting the control of this disease.

**Abstract:**

The selection of porcine reproductive and respiratory syndrome (PRRS) resilient sows has been proposed as a strategy to control this disease. A discrete event-based simulation model was developed to mimic the outcome of farms with resilient or susceptible sows suffering recurrent PRRSV outbreaks. Records of both phenotypes were registered in a PRRSV-positive farm of 1500 sows during three years. The information was split in the whole period of observation to include a PRRSV outbreak that lasted 24 weeks (endemic/epidemic or En/Ep) or only the endemic phase (En). Twenty simulations were modeled for each farm: Resilient/En, Resilient/En_Ep, Susceptible/En, and Susceptible/En_Ep during twelve years and analyzed for the productive performance and economic outcome, using reference values. The reproductive parameters were generally better for resilient than for susceptible sows in the PRRSV En/Ep scenario, and the contrary was observed in the endemic case. The piglet production cost was always lower for resilient than for susceptible sows but showed only significant differences in the PRRSV En/Ep scenario. Finally, the annual gross margin by sow is significantly better for resilient than for susceptible sows for the PRRSV endemic (12%) and endemic/epidemic scenarios (17%). Thus, the selection of PRRSV resilient sows is a profitable approach for producers to improve disease control.

## 1. Introduction

Porcine reproductive and respiratory syndrome (PRRSV) is the most economically significant disease impacting commercial pig production in North America, Europe, and Asia [1]. The various clinical outcomes following PRRSV infection are the consequence of a complex set of interactions between the virus and the host. In sows, PRRSV can cause late-term abortions, prolonged anestrus, an increase in stillborn and mummified piglets, and coughing and respiratory problems, whereas respiratory symptoms and reduced growth performance are observed in young pigs [2,3].

There has emerged a body of evidence that associates host genetics with different outcomes following PRRSV infection [4,5,6]. Moreover, it has also been described that there is a variation in the reproductive performance among sows in response to PRRSV infection, which suggests that there is a scope to select sows for PRRSV resilience [7,8,9,10]. In this case, resilience is defined as the ability of the sow to maintain reproductive performance facing a PRRSV infection. Recently, we demonstrated that, in commercial PRRSV-infected farms, PRRSV resilient sows had less loss of piglets at birth. These sows were phenotyped as resilient if their serum was negative for PRRSV by PCR at seven and 21 d post-vaccination (DPV) with a PRRSV modified live vaccine or as susceptible if their serum was positive by PCR at seven and/or 21 DPV [11]. Nevertheless, the overall efficiency of PRRSV resilient sows remains to be proven for producers. In this sense, modeling could provide new knowledge about the efficiency of PRRSV resilient and susceptible sows in a commercial setting. This information is very difficult to be obtained experimentally due to the necessity to have a complete farm with PRRSV resilient or susceptible sows. In the literature, modeling has been used to predict the effects of future interventions for PRRSV control and its economic impact [12,13].

The goal of this research was to examine whether the use of resilient sows can efficiently improve the reproductive performance in PRRSV endemic-infected farms suffering recurrent PRRSV outbreaks. To achieve this goal, the age of the culled sows, lifelong production, and economic performance were analyzed in simulated farms using PRRSV resilient or susceptible sows.

## 2. Materials and Methods

All experimental procedures were approved by the Ethics Committee for Animal Experimentation of the University of Lleida and performed in accordance with authorization 7700 issued by the Catalan Department of Agriculture, Livestock, Fisheries and Food (Section of biodiversity and hunting).

### 2.1. Farm, Sows, and Data

Data were obtained from a PRRSV-positive commercial farm of 1500 Landrace x Large White sows belonging to a big integration Spanish company (Pinsos del Segre S.A, Lleida, Spain). The farm operated with weekly farrowing batches in an all-in/all-out management system. The routine vaccination program included sow immunization against swine parvovirus, Aujeszky disease virus, swine influenza virus, *Erysipelotrix rhusiopathie*, *Escherichia coli*, and *Clostridium perfringens*. Moreover, piglets were vaccinated against *Mycoplasma hyopneumoniae* (Mhyo) and porcine circovirus type 2 (PCV2) at weaning (three weeks of age).

A randomly sampled subset of 382 sows was classified as PRRSV resilient (*n* = 136) or susceptible (*n* = 246) following the procedure described in Abella et al. (2019) [11]. These sows entered the farm as gilts at the age of seven months (around 130 kg of body weight) at the beginning of March 2016. First, they were allocated in a quarantine unit, where they were vaccinated against swine parvovirus, Aujeszky disease virus, swine influenza virus, *Erysipelotrix rhusiopathie*, PCV2, and Mhyo. Once in the reproduction unit, they were artificially inseminated to meet the weekly reproduction goals of the farm. After each farrowing, the farrowing date; the gestation length; and the number of piglets born alive, stillborn, mummified, and weaned were recorded for three years (March 2016–March 2019). Cross-fostering among litters was maintained as usually practiced in commercial farms. Thus, any swap of piglets between sows was allowed to minimize preweaning mortality.

Data from resilient and susceptible sows were collected from March 2016 to March 2019. The PRRSV health status of the farm was monitored [11], and one PRRSV outbreak was detected in May 2018 following the standard laboratory procedures. Briefly, testing the methods to determine the shedding of this virus included its direct detection by quantitative reverse transcriptase PCR (qRT-PCR) in sick piglets [11]. The PRRSV outbreak was over in November 2018 (24 weeks of duration). The records available for this study are those of backup data recorded until 31 March 2019. The farm health status was classified as endemic or epidemic according to the absence or presence of overt reproductive problems compatible with a PRRSV infection, respectively. The overt reproductive problems (epidemic situation) were based on a significant increase of abortions (>0.3% of the monthly abortion rate) and/or lost piglets (stillborn and mummified) versus the baseline situation (endemic situation) using a probabilistic Poisson-based model to detect PRRSV recirculation with sow production records [14]. Field PRRSV-positive samples, available before the beginning of the study (March 2015), and samples taken during the PRRSV outbreak (May 2018) were sequenced using Sanger technology for ORF5, and a similarity analysis between strains was carried out using the CLC Genomics Workbench 11.0^®^ (Aarhus, Denmark) according to the standard operation procedures of a laboratory that specialized in porcine diseases (http://www.gsplleida.net/es accessed on 10 February 2020). Two strains were classified as different with a similarity lower than 97% in the ORF5 sequence. In this case, the similarity between the two strains collected in March 2015 and May 2018 was 98.2%, suggesting that the resident PRRSV strain was also responsible of the outbreak detected in May 2018.

### 2.2. Simulation Model

The discrete event-based simulation model described in Plà et al. (2018) [15] was used to analyze the productive and economic performance of resilient and susceptible sows in a PRRSV endemic-infected farm suffering recurrent outbreaks. The model was developed in ExtendSim v9 (Imagine That Corporation, San José, CA, USA).

**Figure 1:**
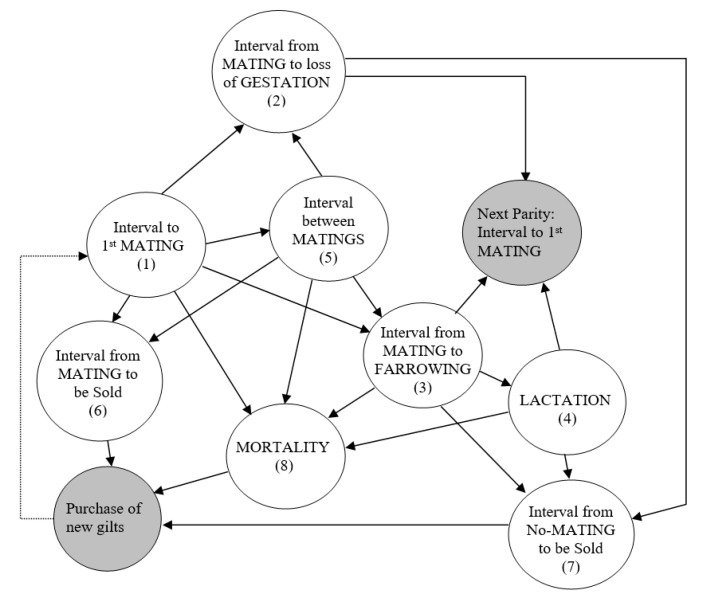
Digraph representing the (first) reproductive cycle of a sow: the states (the eight numbered nodes) and transitions allowed are represented by arrows. Note that there is an entry state representing the purchase of gilts and a link to the next reproductive cycle state (gray color).

The reproductive dynamics of a sow in a crate was described using a Markov model with eight states per reproductive cycle, plus an initial state that corresponded to the time-point when the sow was introduced as a gilt into the farm (Figure 1). Each state is illustrated as a node associated with a time interval distribution, and the arrows connecting the nodes represent a possible change to a different state with a transition probability. The time intervals distributions are shown in Table 1 and Table 2, while the transition probabilities and the rest of the input parameters are presented in Table 3, Table 4 and Table 5. The time intervals for each sow were randomly sampled from the distributions in Table 1 and Table 2 when needed. The transition probabilities were generated as independent real uniforms (0, 1) at any time as a sow moves into a new state according to the rates presented in Table 3 and Table 5. The model assumes that the mortality state had a zero-time interval, which means the immediate replacement of a sow with a gilt ready to mate, and therefore, the quarantine of gilts was not explicitly represented in the model.

**Figure 2:**
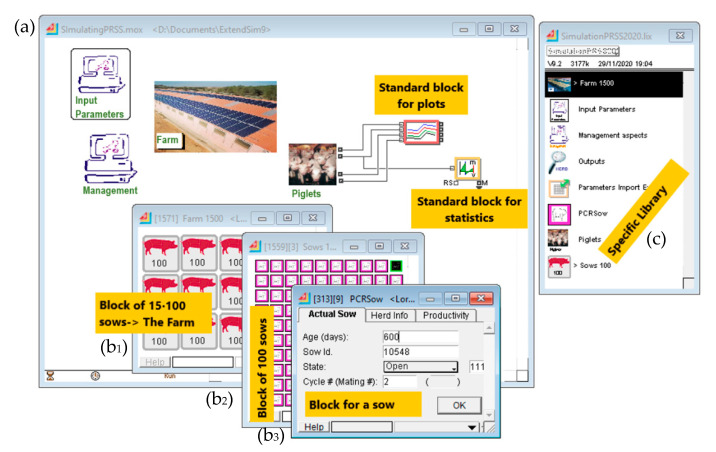
Overview showing (**a**) the model sheet with the main blocks: Input parameters, Management, Piglets, and Farm, besides two standard blocks for plotting and statistics reporting. (**b**) Nested windows illustrate the hierarchy structure of the Farm block (**b_1_**) housing 1500 individual sows grouped into blocks of one hundred (**b_2_**) individual sows (**b_3_**) and (**c**) the library containing the specific blocks of this model.

The features of ExtendSim include graphical modeling by blocks, animation, and the easy generation of reports in Excel or text files for further processing and do not require external interfaces, compilers, or code generators [16,17]. In addition, several blocks can be encapsulated as submodels to improve the model organization and readability. The model used here is organized into four specific blocks: Farm, Input parameters, Management, and Piglets (Figure 2). The Farm block (Figure 2) integrates all farm crates under a unique encapsulated block. The Input Parameters block reads the expected productive performance data of the farm, while the Management block sets the farmer’s management decisions in terms of minimum weaning age and voluntary culling. Voluntary culling refers to the maximum number of parities, maximum number of lost gestations, or maximum number of matings allowed. The lifespan was limited to a maximum of nine reproductive cycles, with a maximum of three matings and one loss of gestation per cycle. The input parameters were calculated from the sow records, as indicated in Section 2.3. The Piglets block simulates the survival rate for piglets at birth and at weaning according to the parameters in Table 4. The piglets are assumed to be weaned on Monday and Thursday at a minimum and maximum of 21 and 27 days old, respectively. All these blocks are held in a library that allows the user to easily customize the size of the farm by adding more or less crates (i.e., sows) into the Farm block. Here, we simulated a 1500-sow farm unit without a nursery, in line with the farm used in Abella et al. (2019) [11] and the average size of a sow farm in Spain [18].

### 2.3. Input Parameters

The input parameters were extracted from the farm described in Section 2.1 either by calculating the sample distributions of several measures of the position (means) or performing calculations of the probability rates, as detailed in Table 6. The data were split by sow (R: resilient and S: susceptible) and farm health status (En: endemic and En/Ep: endemic plus epidemic) in order to simulate four virtual production scenarios (Resilient/En, Resilient/En_Ep, Susceptible/En, and Susceptible/En_Ep). For each production scenario, the distribution of the interval to first mating after weaning, gestation length, interval from mating to culling (excluding gilts), interval from otherwise to culling (excluding gilts), interval from gilt incoming to culling before farrowing, and interval between matings are detailed in Table 1. The time interval distributions from the beginning of gestation to pregnancy loss and the conception rate are given in Table 2 and Table 3, respectively. A pregnancy loss was defined as a loss of a gestation previously confirmed by an ultrasound technique. Thus, the pregnancy loss rate was 7.1%, 7.2%, 9.4%, and 9.3% for first parity sows in the Resilient/En, Resilient/En_Ep, Susceptible/En, and Susceptible/En_Ep subpopulations, respectively. However, for multiparous sows, these rates were 4.9%, 6.1%, 3.3%, and 4.9% for the Resilient/En, Resilient/En_Ep, Susceptible/En, and Susceptible/En_Ep subpopulations, respectively.

The number of piglets born alive and weaned per litter by parity, sow status, and farm health status are shown in Table 4. Sow culling included both voluntary (sows sent to abattoir or slaughtered) and involuntary (dead or euthanized) events. The culling rate by parity and sow health status is detailed in Table 5. The probability of R sows being culled due to involuntary and voluntary decisions was 50.0% in the En scenario, whereas this probability for culled R sows was 67.5% and 32.5% for involuntary and voluntary decisions in En/Ep, respectively. In contrast, the probability of S sows being culled due to involuntary or voluntary decisions was 57.6% and 42.4% in En and 70.3% and 29.7% in En/Ep, respectively. The same reproductive management was set for the four simulated farms.

Each of the four virtual farms was simulated 20 times. The outcome of each simulation was the performance of all sows in the farm over a time horizon of 12 years. To assure the independence of the observations and to avoid perturbations due to the transient period, only the last year of each simulation was considered in the calculations. The results for each virtual farm were expressed as the mean (SD) of the 20 simulation runs.

### 2.4. Economic Modeling

The economic setting was based on the local market conditions in Spain in 2019 [19] (Table 7). The annual gross margin (GM) was calculated as the difference between the total annual revenue minus the total annual cost. The revenue is the annual income due to the sale of weaned piglets and culled sows sent to the abattoir. The total annual cost was calculated from four components: replacement, feeding, mating cost, and other costs. The replacement cost is the expense of purchasing the gilts that replace culled sows. The annual feeding cost is calculated from the accumulated daily feed consumption of all sows according to their reproductive state (pregnancy or lactation). The same prices were assumed over the whole period, while creep feeding for piglets was not taken into account. The annual mating cost accounted for the cost of the artificial inseminations performed during a year. Other costs included the management, administration, veterinary, and drug and vaccine expenses.

### 2.5. Comparison of Virtual Farms

The simulation outcome was managed and analyzed with ExtendSim (Imagine That Corporation, San José, CA, USA) and JMP (SAS Inst. Inc., Cary, NC, USA). Each replicate of every virtual farm was used as the experimental unit. The significance level was set at 0.05. Productive and economic outcomes were presented for each virtual farm (Resilient/En, Resilient/En_Ep, Susceptible/En, and Susceptible/En_Ep). The Shapiro-Wilk test was used to test for normality in continuous variables, while Levene’s test was used to test for the homogeneity of variances. The association of the four virtual farms with non-normal and normal continuous variables was analyzed using the Wilcoxon test (with the Mann–Whitney *U* test to compare each pair of values) and the ANOVA test (with Tukey’s Honest Significant Difference to compare each pair of values), respectively.

## 3. Results

### 3.1. Production Performance Outcome for Resilient and Susceptible Sows

**Figure 3:**
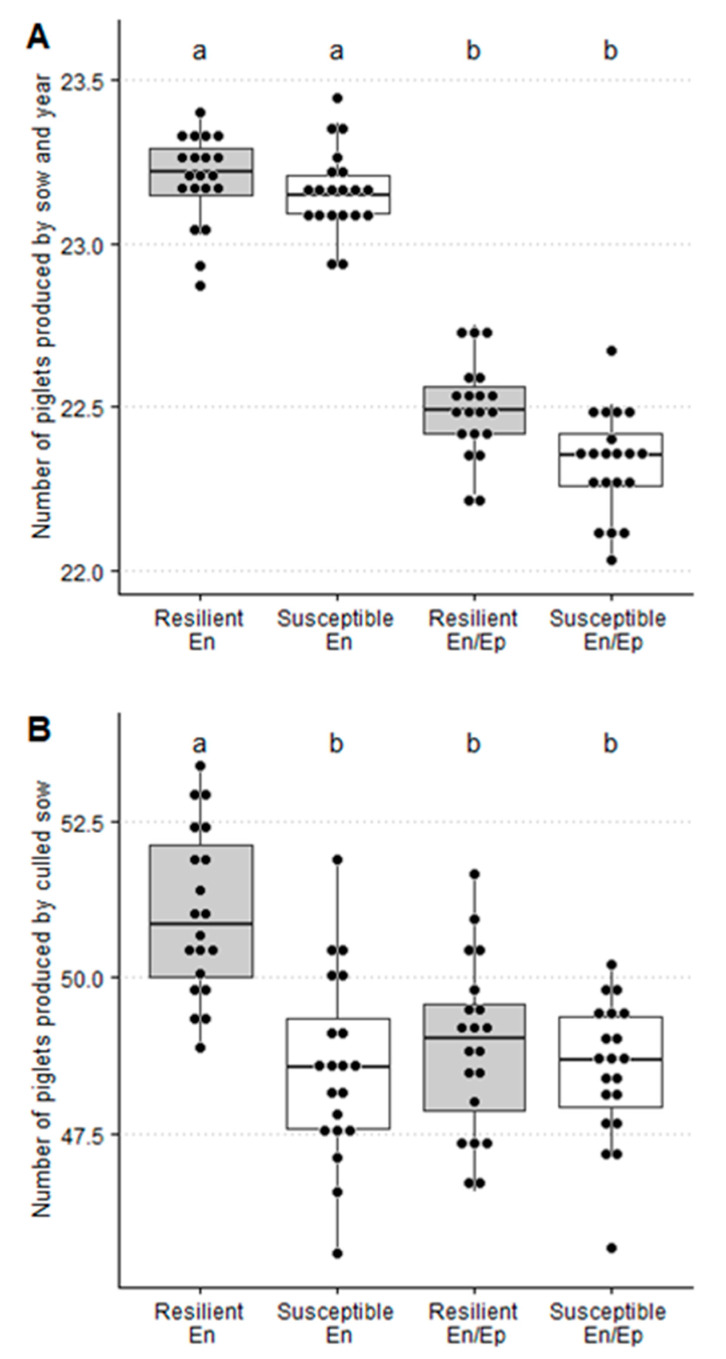
Number of piglets produced by sow and year (**A**), and the number of piglets produced by culled sow (**B**) for porcine reproductive and respiratory syndrome (PRRSV) resilient and susceptible sows in a PRRSV endemic-infected farm not suffering (En) or experiencing (En/Ep) one epidemic PRRSV outbreak with an average duration of 24 weeks within three years. Bars with different superscripts showed statistically significant differences (*p* < 0.05).

**Figure 4:**
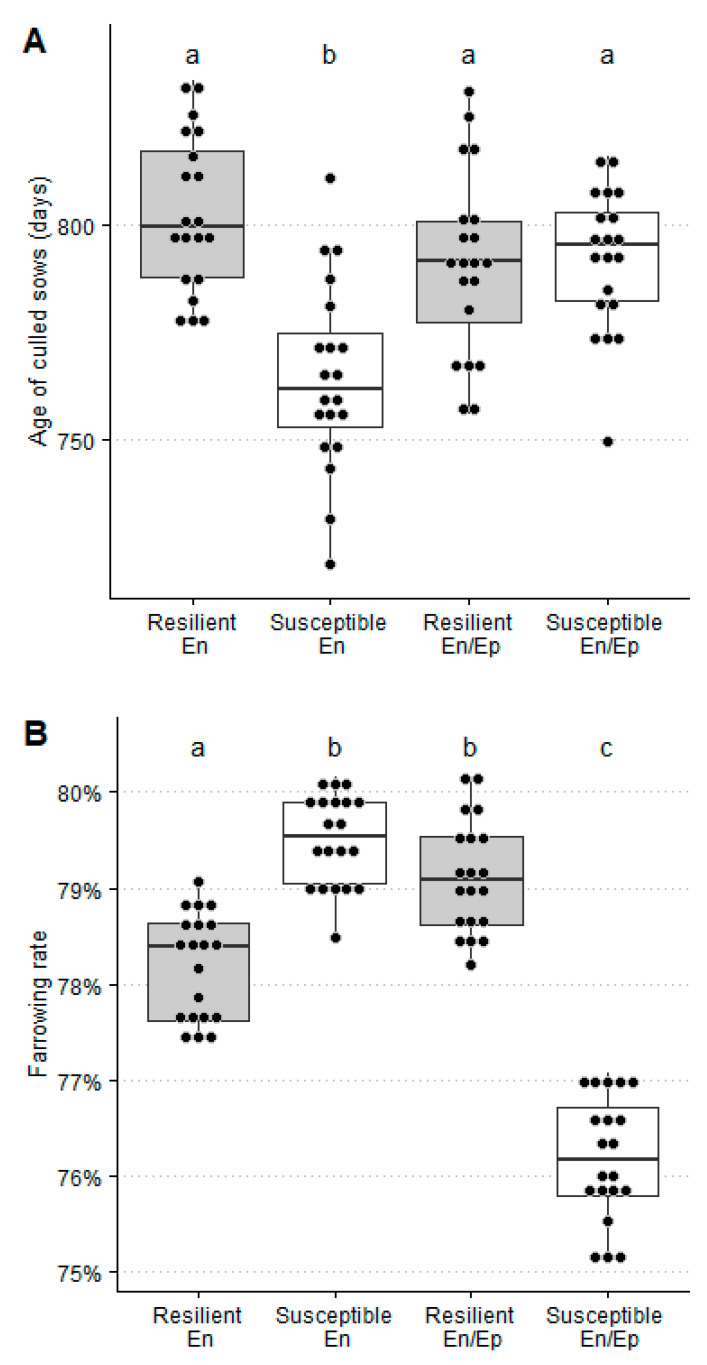
Average age of culled sows (days) on the farm (**A**) and farrowing rate (%) (**B**) for PRRSV resilient and susceptible sows in a PRRSV endemic-infected farm not suffering (En) or experiencing (En/Ep) one epidemic PRRSV outbreak with an average duration of 24 weeks within three years. Bars with different superscripts showed statistically significant differences (*p* < 0.05).

In a PRRSV endemic scenario, the repetition rate (7.9% ± 0.5%), number of artificial inseminations by mating (3.14 ± 0.02), the age of culled sows (764 ± 22 days), and the number of piglets produced by culled sow (48.6 ± 1.5 piglets) were significantly lower for susceptible than for resilient sows (10.2% ± 0.5%, 3.19% ± 0.02%, 803 ± 18 days, and 51.0 ± 1.3 piglets, respectively) whereas the farrowing rate and replacement rate were significantly higher for susceptible (79.5% ± 0.5% and 48.1% ± 1.7%) than for resilient ones (78.7% ± 0.5% and 45.4% ± 1.6%, respectively). Moreover, no significant differences were observed between resilient and susceptible sows for the loss of gestation rate, farrowing interval, and number of piglets produced by sow and year (Table 8 and Figure 3 and Figure 4).

In a PRRSV epidemic/endemic scenario, the loss of gestation rate (6.4% ± 0.4%) and farrowing rate (76.2% ± 0.6%) were significantly lower for susceptible than for resilient sows (8.0% ± 0.5% and 79.1% ± 0.6%), whereas the repetition rate (12.3% ± 0.5%) and number of artificial inseminations by mating (3.26 ± 0.03) were significantly higher for susceptible than for resilient ones (9.7% ± 0.5% and 3.20 ± 0.02, respectively). Moreover, no significant differences were observed between resilient and susceptible sows for the replacement rate, age of culled sows, farrowing interval, number of piglets produced by culled sow, and number of piglets produced by sow and year (Table 8 and Figure 3 and Figure 4).

In summary, the reproductive sow parameters were generally better for resilient than for susceptible sows in the PRRSV En/Ep scenario and, on the contrary, was observed in the case of the PRRSV endemic scenario, but the sow herd retention and number of piglets by culled sow were significantly better for resilient than for susceptible sows in the PRRSV endemic scenario.

### 3.2. Economic Performance Outcome for Resilient and Susceptible Sows (all the Parameters in Euros)

**Figure 5:**
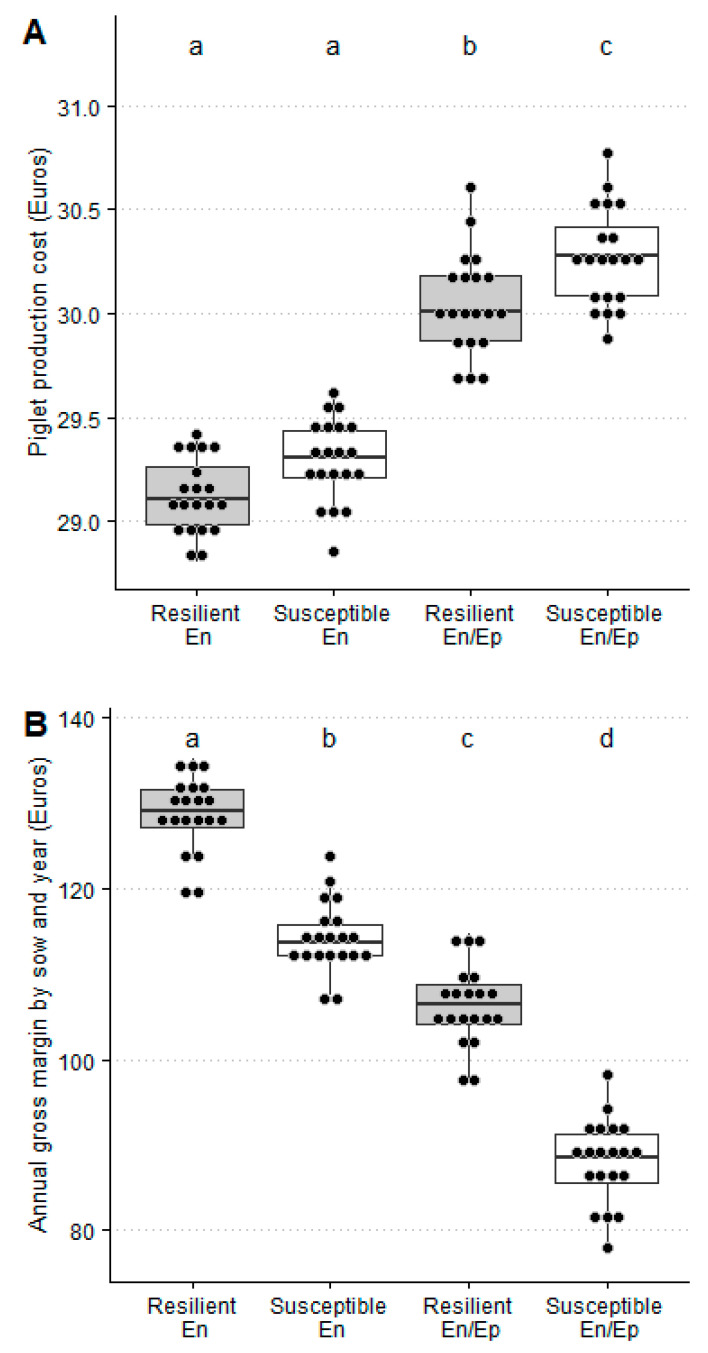
Piglet production cost (Euros) (**A**) and gross margin (**B**) (Euros by year and sow) for PRRSV resilient and susceptible sows in a PRRSV endemic-infected farm not suffering (En) or experiencing (En/Ep) one epidemic PRRSV outbreak with an average duration of 24 weeks within three years. Bars with different superscripts showed statistically significant differences (*p* < 0.05).

In a PRRSV endemic scenario, sow sales (44.69 ± 1.57), artificial insemination cost by sow and year (28.25 ± 0.18), and annual gross margin by sow and year (114.33 ± 4.12) were significantly lower for susceptible than for resilient sows (54.92 ± 1.91, 28.75 ± 0.14, and 128.76 ± 4.36), whereas the purchase cost of replacement by sow and year (59.59 ± 2.1) and feed cost by sow and year (257.65 ± 0.15) were significantly higher for susceptible than for resilient ones (56.33 ± 1.96 and 257.43 ± 0.19, respectively). Moreover, no significant differences were observed between resilient and susceptible sows for the piglet production cost and piglet sales (Table 9 and Figure 5).

In a PRRSV epidemic/endemic scenario, sow sales (42.70 ± 1.30) and the annual gross margin by sow and year (88.03 ± 4.94) were significantly lower for susceptible than for resilient (55.59 ± 2.10 and 106.34 ± 4.63) sows, whereas the artificial insemination cost by sow and year (29.35 ± 0.24) and piglet production cost (30.27 ± 0.24) were significantly higher for susceptible than for resilient ones (28.81 ± 0.21 and 30.04 ± 0.24, respectively). Moreover, no significant differences were observed between resilient and susceptible sows for the purchase cost of replacement by sow and year, feed cost by sow and year, and piglet sales (Table 9 and Figure 5).

In summary, the piglet production cost was significantly higher for susceptible (1%) than for resilient sows only in the PRRSV endemic/epidemic scenario (Figure 5), and the annual gross margin by sow and year was significantly higher in resilient than susceptible sows in both PRRSV scenarios (Figure 5) when taking into account the reference values for the year 2019 (Table 7).

## 4. Discussion

A PRRSV control is probably one of the most important challenges in swine medicine. A good control of this disease must take into account external and internal biosecurity, good husbandry protocols, and vaccination programs for sows and/or piglets. Unfortunately, it is difficult to design effective PRRSV vaccines due to the high virus variability and the difficulty to generate a protective immune response in a short period of time [20]. Thus, new tools are urgently needed to improve the control of this disease under field conditions [3]. Resilient pigs could be an option, because these animals will show less clinical signs and likely shed fewer viruses after PRRSV infection [21], with a subsequent improvement in health and productive performance within and between herds [5]. PRRSV resilient pigs can be directly obtained by gene editing [22] or selected using genomic approaches, including specific genetic markers [23,24,25,26] or ad hoc phenotypes [11]. The phenotyping procedure used in this research work can be carried out under field conditions, because a PRRSV RT-PCR is a common diagnostic technique in many veterinary laboratories. Nevertheless, it remains unclear whether the use of resilient pigs is profitable for producers. To answer this question, we assessed the outcome of the sows identified as resilient or susceptible following Abella et al. (2019) [11] under different PRRSV scenarios. A simulation is a methodology useful to study complex problems with a huge number of interactions, particularly when real-life experiments are very difficult, costly, or even impossible to be carried out [27]. In this study, resilient and susceptible sows were compared using the results of virtual farms simulated with experimental data. The output of such simulations could help to delineate the scope of this approach and eventually support veterinarians and experts in its application [12]. Finally, vaccine-based interventions were not applied in this farm, and the farm management and biosafety issues were the same for resilient and susceptible sows to make comparisons feasible. Thus, our study cannot address the suitability of other approaches to control this disease. Moreover, we cannot provide epidemiologic information, such as PRRSV prevalence, in resilient versus susceptible sows in the simulated farms after applying this strategy.

The input parameters and the assumptions of the model are critical issues in the simulation. Here, all input parameters were obtained from a PRRSV endemically infected farm in which sows were phenotyped for resilience and suffered a PRRSV outbreak [11]. This epidemiological situation, i.e., a combination of PRRSV endemic and epidemic periods, is highly frequent in areas where the prevalence of this disease is extremely high [28,29]. Thus, the input parameter distributions were not obtained through a stochastic simulation, as in other PRRSV economic studies [12,30], but using experimental data. We believe that this approach is robust enough to decipher the potential differences between phenotypes (resilient versus susceptible), mimicking the reality in high-density pig areas. It must be highlighted, however, that the productive and economic results are correct for the input parameters, prices, and costs used. In any other situation, the results associated with the resilience might differ due to the virulence of the PRRSV strain, the duration of the outbreaks, the presence of coinfections [31], or prices and costs, which can show a strong variation between countries and even between individual farms [32,33]. Nevertheless, it does not invalidate the comparison between resilient and susceptible sows, since all of these constraints are expected to affect both phenotypes without any bias in our simulation setting.

Our results indicate that sows show a better reproductive outcome in the PRRSV endemic than in the PRRSV endemic/epidemic scenario. This is in line with the expected results for a virus that affects sows during the gestation period [34]. The differences between resilient and susceptible sows must be discussed when taking into account the reproductive outcome for each epidemiological scenario. Although resilient sows showed the highest losses of gestation in both PRRSV epidemiological scenarios, the losses occurred at different pregnancy time points. Thus, while in resilient sows, losses happened mostly at the beginning of gestation, in susceptible sows, these took place towards the end (Table 2). This finding is in agreement with the experiment described by Abella et al. (2019) [11], where the largest difference between resilient and susceptible sows was observed for mummified piglets during a PRRSV outbreak, and with the fact that increased mummified piglets is the most identifiable clinical outcome in an acute PRRSV outbreak in sows. Note, however, that the variable we used for measuring the losses of the gestation rate is not comparable with the abortion rate, commonly used in many studies addressing a PRRSV economic analysis [12,13]. The abortion rate only accounts for losses in the fetal stage (≥35–40 days of gestation), whereas the losses of gestation rate includes both embryonic and fetal losses. The repetition rate showed a different pattern between resilient and susceptible sows depending on the PRRSV epidemiological scenario, with resilient sows showing lower values than susceptible sows in PRRSV En/Ep and higher values in the PRRSV En scenario. Finally, the other reproductive outcome is the number of piglets produced by culled sows. This parameter measures the sow herd retention at the farm level. In this experiment, the value obtained for resilient sows was better than for susceptible ones mainly in the endemic scenario. This parameter has not been sufficiently described in the literature, but an optimum sow herd retention decreases the involuntary herd turnover, maintaining the planned genetic progress, the parity profile in the farm, and, as a consequence, the overall performance of the herd [35]. This parameter has recently started being considered as a criterion in pig breeding companies [36]. Finally, no difference was observed between resilient and susceptible sows for the number of piglets weaned by sow and year in any of the two PRRSV epidemiological scenarios. This result, although surprising, can be explained because of cross-fostering, which was allowed among sows from the same batch regardless of their phenotype. Cross-fostering is a common practice to equalize litter sizes, thereby probably decreasing the difference between phenotypes in terms of weaned piglets. Thus, the differences observed between phenotypes could probably be lower than the real difference due to the homogenizing effect on the number of weaned piglets by sow due to cross-fostering practices.

The total cost of piglet production can be divided into feed cost; mating cost; gilt replacement cost; and other costs, including drug, veterinary, and vaccine costs. We assumed that the two phenotypes had the same other costs, although the veterinary cost may be higher in susceptible sows after a PRRSV outbreak [30] due to the treatment of secondary infections. Thus, for this component of the cost, we are probably underestimating its value for susceptible sows. We found that the piglet production cost is 3% lower in a PRRSV endemic than in a PRRSV endemic/epidemic scenario, which is in line with the results from theoretical studies on the cost and impact of associated control measures against PRRSV [12]. Interestingly, in a PRRSV endemic/epidemic scenario, the piglet production cost is significantly lower for resilient (1%) sows as compared to susceptible sows, which confirms breeding for resilience as an interesting strategy for PRRSV control [13]. Moreover, as a proactive prevention measure, compared to reactive disease treatments, resilience is widely accepted amongst consumers [37]. Breeding for resilience is perceived by the public as a more natural approach than vaccines to control diseases, thereby adding more value to the pig chain [38].

Resilient sows had greater annual gross margins. The annual gross margin (GM) by sow was 12% (14.40 euros) and 17% (18.30 euros) greater for resilient than for susceptible sows in the PRRSV En and En/Ep scenarios, respectively. Most of this difference (10 and 13 euros, respectively) were from greater sow sales to slaughterhouses resulting from a reduction in involuntary losses. The GM was calculated on a weaned piglet basis. It was not considered that piglets from resilient sows will probably also develop better throughout the growing period. For this reason, the GM calculated here is likely underestimated with respect to the values obtained in cases where the impact of PRRSV is measured until the end of the growing phase [13].

## 5. Conclusions

Resilient sows are more profitable, because they show an enhanced age of culled sows, lifelong production, and economic performance. Identifying PRRSV resilience sows is a profitable strategy in PRRSV endemic-infected farms suffering periods of epidemic outbreaks.

## Figures and Tables

**Table 1:** Time interval distributions, in days, obtained from data in the included a farm from March 2016 to March 2019. Distributions were obtained by splitting the observation period into an endemic period (En) (from March 2016 to May 2018) and endemic and epidemic period (En/Ep) (from March 2016 to March 2019). *N*(x,y) means a normal distribution, where x and y are the mean (in bold) and standard deviation of the distribution, respectively. log*N*(x,y) means a log normal distribution, where x and y are the mean and standard deviation of the distribution, respectively.

	En		En/Ep	
	Resilient	Susceptible	Resilient	Susceptible
Interval to first mating after weaning (days)				
Gestation	*N*(116,1.8)	*N*(116,1.8)	*N*(116,1.8)	*N*(116,1.8)
Length (days) Interval from mating to culling (not including gilts) (days)	*N*(61,29.4)	*N*(56,34.0)	*N*(57,28.9)	*N*(55,32.6)
Interval from otherwise to culling (not including gilts) (days)	*N*(26,28.8)	*N*(26,32.5)	*N*(21,25.1)	*N*(21,28.8)
Interval from gilt incoming to culling before farrowing (days)	log*N*(3.82,0.67)	log*N*(3.82,0.67)	log*N*(3.82,0.67)	Log*N*(3.82,0.67)

The interval between matings was considered the same for all scenarios and represented by a mixture of three normals: X_1_ ≈ *N*(22,4.5), X_2_ ≈ *N*(44,3.8), and X_3_ ≈ *N*(70,8.6) and probability of each: *p*_1_ = 79%, *p*_2_ = 15%, and *p*_3_ = 6% capturing the heat detection errors on a farm.

**Table 2:** Time interval distributions from the beginning of gestation to time of pregnancy losses from registered data from March 2016 to March 2019. Different distributions were obtained when splitting the observation period into an endemic period (En) (from March 2016 to May 2018) and endemic and epidemic period (En/Ep) (from March 2016 to March 2019). The resulting time interval distributions fit a mixture of two normal distributions. *N*(x,y) means a normal distribution, where x and y are the mean and standard deviation of the distribution, respectively. Thus, for example, for resilient sows in the endemic status, the two normal were X_1_ ≈ *N*(33,10.6) and X_2_ ≈ *N*(97,10.1), with a probability of *p*_1_ = 50% and *p*_2_ = 50%, respectively.

	En		En/Ep	
	Resilient	Susceptible	Resilient	Susceptible
Mean 1	33	26	32	27
Sd 1	10.6	7.2	10.6	7.3
Probability 1	50%	26%	38%	25%
Mean 2	97	99	100	100
Sd 2	10.1	14.0	9.7	13.5
Probability 2	50%	74%	62%	75%

**Table 3:** Conception rates obtained from the data in the included farm from March 2016 to March 2019. Rates were obtained by splitting the observation period into an endemic period (from March 2016 to May 2018) and endemic and epidemic period (from March 2016 to March 2019).

	En		En/Ep	
	Resilient	Susceptible	Resilient	Susceptible
Mating 1	0.903	0.908	0.902	0.915
Mating 2	0.816	0.829	0.808	0.774
Mating > 3	0.889	0.786	0.857	0.848

**Table 4:** Number of piglets born alive (NBA) and number of weaned piglets per litter from the data in the included farm from March 2016 to March 2019. Both parameters were obtained by splitting the observation period into an endemic period (from March 2016 to May 2018) and endemic and epidemic period (from March 2016 to March 2019). sd: standard deviation.

		En				En/Ep			
		Resilient		Susceptible		Resilient		Susceptible	
	Parity	mean	sd	mean	sd	mean	sd	mean	sd
NBA	1	10.52	2.66	10.91	3.12	10.52	2.65	10.91	3.12
	2	11.22	3.49	11.81	3.50	11.13	3.60	11.81	3.50
	3	11.61	3.02	11.62	3.18	11.95	2.98	11.71	3.42
	4	12.00	3.36	12.49	3.22	11.91	3.22	12.31	3.49
	5	12.41	3.13	11.98	2.89	12.29	3.06	12.03	3.09
	6	13.50	5.04	12.52	3.75	12.28	3.72	12.20	3.40
	>7	13.50	1.73	11.22	2.90	10.63	3.31	11.10	2.96
Weaned piglets per litter	1	9.95	0.61	9.94	0.53	9.93	0.64	9.94	0.53
	2	10.07	0.78	10.23	0.81	10.07	0.77	10.23	0.81
	3	10.54	0.96	10.39	0.74	10.48	1.01	10.31	1.61
	4	10.35	0.69	10.64	1.02	10.13	1.16	10.42	1.34
	5	10.27	0.51	10.23	0.54	10.28	0.99	10.41	0.81
	6	10.67	0.82	10.48	0.68	9.83	1.43	10.06	1.42
	>7	10.23	0.64	9.69	0.69	10.23	1.14	9.69	1.39

**Table 5:** Culling rates (%) of involuntary (mortality and euthanized at farm by humanitarian reasons) and voluntary (sows sent to abattoir or slaughtered sows) decisions from the data in the included farm from March 2016 to March 2019. Rates were obtained by splitting the sows according to the phenotypes of resilient and susceptible.

	Resilient	Susceptible
Gilt culled before mating	3.60	3.88
Gilt culled before farrowing	5.22	6.05
Sow parity 1	7.38	3.59
Sow parity 2	7.21	10.23
Sow parity 3	3.49	7.74
Sow parity 4	9.09	7.29
Sow parity 5	17.02	19.40
Sow parity > 6	17.02	16.13

**Table 6:** Definition of the procedure to calculate several rates either as input parameters (calculation from farm data records) or output parameters (calculation from simulated records).

Productive Parameter	Calculus Procedure
Conception rate	(#Pregnancy losses * + #Farrowings)/#matings
Culling rate per parity, *n*	#Culled sows in parity *n*/#Sows in parity, *n*
Repetition rate	#matings - #1st matings/#matings
Loss of gestation rate	#Pregnancy losses/(#Pregnancy losses + #Farrowings)
Replacement rate	#Culled sows/#Sows
Farrowing rate	#Farrowings/(#Pregnancy losses + #Farrowings)

* A pregnancy loss was defined as the loss of a gestation previously confirmed by an ultrasound technique. # means the number total of events.

**Table 7:** Input parameters for calculating the economic outcome in a typical Northeast Spanish sow farm (DARP, 2020).

Parameter	Value
Price per sow slaughtered	186.00 €
Price of replacement gilt	124.00 €
Price per piglet (6 kg)	32.30 €
Feed price/ton	250.00 €
Feed consumption per sow and day (open gestation)	2.5 kg
Feed consumption per sow and day (lactation)	5.5 kg
Insemination price (two doses)	9.00 €
Veterinary, management, drugs, vaccines, and housing per sow and year	333.00 €

**Table 8:** Annual production parameters for each virtual farm by taking into account the phenotype of the sow (Resilient or Susceptible) and the porcine reproductive and respiratory syndrome (PRRSV) epidemiological scenario (Endemic versus Endemic and Epidemic). The epidemic phase corresponds to one PRRSV outbreak that lasted 24 weeks in duration within three years.

Virtual Farm		RR (%)		Rep (%)		LGR (%)		FI		AI	
Phenotype	PRRSV	Mean	sd	Mean	sd	Mean	sd	Mean	sd	Mean	sd
Resilient	En/Ep	46.0% ^b^	1.7%	9.7% ^c^	0.5%	8.0% ^a^	0.5%	156.6 ^a^	0.9	3.20 ^b^	0.02
Susceptible	En/Ep	45.9% ^b^	1.4%	12.3% ^a^	0.5%	6.4% ^b^	0.4%	156.9 ^a^	0.9	3.26 ^a^	0.03
Resilient	En	45.4% ^b^	1.6%	10.2% ^b^	0.5%	5.9% ^c^	0.3%	155.3 ^b^	0.9	3.19 ^b^	0.02
Susceptible	En	48.1% ^a^	1.7%	7.9% ^d^	0.5%	5.6% ^c^	0.3%	154.9 ^b^	0.8	3.14 ^c^	0.02

RR: replacement rate, Rep: repetitions rate, LGR: Loss of gestation rate, FI: interval between farrowings, and AI: number of artificial inseminations by mating. sd: standard deviation. Values in each column (a, b, c, d) with different superscripts showed statistically significant differences (*p* < 0.05).

**Table 9:** Main economic parameters by sow and year for each virtual farm by taking into account the phenotype of the sow (Resilient or Susceptible) and the PRRSV epidemiological scenario (Endemic versus Endemic and Epidemic). The epidemic phase corresponds to one PRRSV outbreak of 24 weeks in duration every three years.

Virtual Farm		Sow Sales (€)		Piglets (€)		Gilts (€)		Feed (€)		Mating (€)	
Phenotype	PRRSV	Mean	sd	Mean	sd	Mean	sd	Mean	sd	Mean	sd
Resilient	En/Ep	55.59 ^a^	2.10	726.4 ^b^	4.74	57.0 ^b^	2.15	256.9 ^c^	0.21	28.8 ^b^	0.2
Susceptible	En/Ep	42.70 ^b^	1.30	721.3 ^b^	4.98	56.9 ^b^	1.74	256.7 ^c^	0.16	29.4 ^a^	0.2
Resilient	En	54.92 ^a^	1.91	749.3 ^a^	4.48	56.3 ^b^	1.96	257.4 ^b^	0.19	28.7 ^b^	0.1
Susceptible	En	44.69 ^b^	1.57	748.1 ^a^	4.11	59.6 ^a^	2.10	257.6 ^a^	0.15	28.3 ^c^	0.2

Sow sales: Economic value of culled sows by sow and year, Piglets: economic value of weaned piglets by sow and year, Gilts: purchase cost of replacement by sow and year, Feed: feed cost by sow and year, and Mating: artificial insemination cost by sow and year. sd: standard deviation. Values in each column (a, b, c) with different superscripts showed statistically significant differences (*p* < 0.05).

## Data Availability

Restrictions apply to the availability of these data. Data was obtained from Pinsos del Segre SA and are available from the authors with the permission of Pinsos del Segre SA.
